# Bioactivation and Regioselectivity of Pig Cytochrome P450 3A29 towards Aflatoxin B_1_

**DOI:** 10.3390/toxins8090267

**Published:** 2016-09-12

**Authors:** Jun Wu, Ruohong Chen, Caihui Zhang, Kangbai Li, Weiying Xu, Lijuan Wang, Qingmei Chen, Peiqiang Mu, Jun Jiang, Jikai Wen, Yiqun Deng

**Affiliations:** Guangdong Provincial Key Laboratory of Protein Function and Regulation in Agricultural Organisms, College of Life Sciences, South China Agricultural University, Guangzhou 510642, China; wujun@scau.edu.cn (J.W.); Ruohongchen@hotmail.com (R.C.); zhangcaihui11@gmail.com (C.Z.); kangboli@gmail.com (K.L.); grace87330@163.com (W.X.); wlj322@scau.edu.cn (L.W.); chenqingmei@scau.edu.cn (Q.C.); mpeiqiang@scau.edu.cn (P.M.); jiangjun@scau.edu.cn (J.J.); jkwen@scau.edu.cn (J.W.)

**Keywords:** cytochrome P450, CYP3A29, AFB_1_, bioactivation, regioselectivity

## Abstract

Due to unavoidable contaminations in feedstuff, pigs are easily exposed to aflatoxin B_1_ (AFB_1_) and suffer from poisoning, thus the poisoned products potentially affect human health. Heretofore, the metabolic process of AFB_1_ in pigs remains to be clarified, especially the principal cytochrome P450 oxidases responsible for its activation. In this study, we cloned CYP3A29 from pig liver and expressed it in *Escherichia coli*, and its activity has been confirmed with the typical P450 CO-reduced spectral characteristic and nifedipine-oxidizing activity. The reconstituted membrane incubation proved that the recombinant CYP3A29 was able to oxidize AFB_1_ to form AFB_1_-exo-8,9-epoxide in vitro. The structural basis for the regioselective epoxidation of AFB_1_ by CYP3A29 was further addressed. The T309A mutation significantly decreased the production of AFBO, whereas F304A exhibited an enhanced activation towards AFB_1_. In agreement with the mutagenesis study, the molecular docking simulation suggested that Thr309 played a significant role in stabilization of AFB_1_ binding in the active center through a hydrogen bond. In addition, the bulk phenyl group of Phe304 potentially imposed steric hindrance on the binding of AFB_1_. Our study demonstrates the bioactivation of pig CYP3A29 towards AFB_1_ in vitro, and provides the insight for understanding regioselectivity of CYP3A29 to AFB_1_.

## 1. Introduction

Aflatoxins are secondary metabolites mainly produced by *Aspergillus* *flavus* and *Aspergilus parasiticus* [1]. They are highly toxic and carcinogenic to animals and humans. Aflatoxin infection is believed to induce hepatocellular carcinoma [2]. Aflatoxin B_1_ (AFB_1_) is the most toxic and widely distributed of all identified aflatoxins [3,4]. AFB_1_ is not toxic in its native form, but highly activated when converted to electrophilic AFB_1_ exo-8,9-epoxide (AFBO) in vivo [5,6]. AFBO is highly reactive and easily intercalates into DNA, resulting in the genotoxicity of AFB_1_ [7]. Extensive research has confirmed that CYP3A4 and CYP1A2 play central roles in the bioactivation of AFB_1_ in human [8,9,10], though there remain some controversies about their relative dominance during this process [10,11,12,13]. Compared to CYP3A4, CYP1A2 is believed to have a higher affinity to AFB_1_ and to activate the carcinogen at relative lower substrate concentrations [11,12]. However, some researchers still think CYP3A4 plays a more important role in AFB_1_ activation based on the following experimental facts: (i) CYP3A4 is far more abundant than CYP1A2 in the human liver [14]; (ii) CYP3A4 transforms AFB_1_ into exo-8,9-epoxide and a detoxification product AFQ_1_ [15]; and (iii) CYP1A2 metabolizes AFB_1_ into detoxification product AFM_1_, exo- and endo-8,9-epoxides [15], in which endo-8,9-epoxide is not biologically active [16,17]. As the most abundant CYP in the human liver and also the major enzyme responsible for the metabolism of many exogenous and endogenous compounds, CYP3A4 has been studied extensively [18,19,20,21]. Some CYP3A4 orthologs in other species or tissues have been identified and proved to play the same important roles as human CYP3A4 in AFB_1_ activation. For instance, CYP3A37 in the turkey liver [22], CYP3A6 in the rabbit liver [23] and CYP3A enzymes in the human lung [24] have been shown to be enzymes that activate AFB_1_ into AFBO. Besides CYP1A2 and CYP3A4, it has been reported that human CYP2A6, CYP2A13, and CYP2B7 are also active in converting AFB_1_ to active metabolites [8,9,25,26,27], which shows that humans have a large capacity to activate the toxin. Because of the low content in the liver [28], it is unlikely that CYP2A6 and CYP2B6 have a major contribution to AFB_1_ activation in vivo. CYP3A5 is another important human CYP3A isoform, which shares 85% amino acid sequence identity with CYP3A4 [29] and account for approximately 30%–40% of the total hepatic 3A [30]. Different to CYP1A2 and CYP3A4, CYP3A5 largely transforms AFB_1_ into AFBO [29,31], while its transformation rate is notably lower than the others [11,29].

Pigs are indispensable domestic animals in stockbreeding, and their products have a long successful history of providing tremendous contributions to human nutrition and health. In addition, although rodents are the species generally used for metabolic and pharmacological studies, pigs could provide closer reflections of the metabolic mechanism of drugs and other xenobiotics, and consequently have been attracting increasing attention [32]. As one of the domestic animals that are most raised and consumed in China, pigs are easily exposed to AFB_1_ and suffer from poisoning due to aflatoxin-contaminated feedstuff [33]. However, the metabolic pathway of AFB_1_ in pigs, especially the principal P450 oxidases responsible for the activation of AFB_1_, has yet to be addressed in research.

CYP3A29 is one of the pig CYP3A isoforms, and it has been reported to have a major impact in total CYP3A activity in pig liver microsomes [34]. Specifically, pig CYP3A29 has been shown to have similarity to human CYP3A4 in tissue distribution, testosterone oxidation kinetics, and ketoconazole inhibitory kinetics [34].

In this study, we demonstrated that the recombinant CYP3A29 expressed in *E. coli* was capable of transforming AFB_1_ into AFBO. According to molecular docking prediction and the related study reports [35,36], we selected Ser119, Phe304, and Thr309 as potentially important residues that may be involved in the interaction of CYP3A29 with AFB_1_. To gain further insights into the regioselectivity of CYP3A29 towards AFB_1_, we purified S119A, F304A, and T309A mutants and compared their activities in AFB_1_ epoxidation. Based on the results of mutant activity, further molecular docking showed that Thr309 played a key role by forming a hydrogen bond with AFB_1_; Phe304 caused steric hindrance to AFB_1_ and Ser119 contributed to polar interaction. To our best knowledge, this is the first report about pig CYP that is involved in the metabolic activation of AFB_1_, and this work exhibits the difference of regioselectivity of AFB_1_ epoxidation between CYP3A29 and CYP3A4.

## 2. Results

### 2.1. Activation of AFB_1_ by CYP3A29

**Figure 1:**
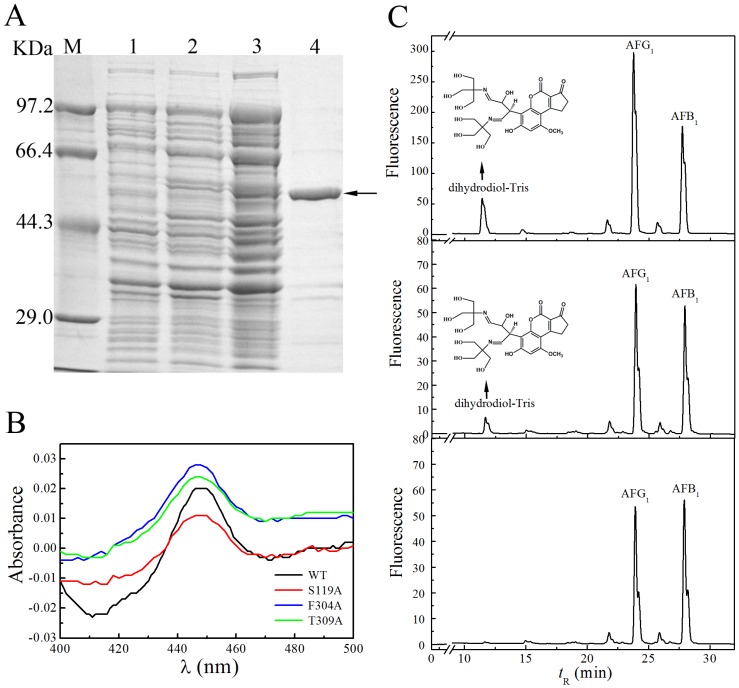
Purification, CO difference spectra, and AFB_1_ oxidation activity of recombinant pig CYP3A29. (**A**) Expression and purification of CYP3A29 in *E. coli* DH5α. The band around 60 kDa is the protein of interest (arrow). M: protein marker; Lane 1: non-IPTG induced total cell lysates; Lane 2: IPTG-induced total cell lysates; Lane 3: the solubilized membrane components; Lane 4: purified recombinant pig CYP3A29. (**B**) Fe^2+^ ∙ CO vs. Fe^2+^ difference spectra of CYP3A29 and the mutants. The spectra were recorded at 25 °C and in 100 mM Tris∙HCl buffer (pH 7.4) containing 10 mM CHAPS, 20% glycerol, and 1 mM EDTA. (**C**) Top: the HPLC chromatogram of AFB_1_ metabolized by quail liver microsomes; Middle: HPLC chromatogram of AFB_1_ metabolized by recombinant CYP3A29; Bottom: CYP3A29 was inhibited by 5 μM ketoconazole and no metabolite formed. The dihydrotriol-tris is an indirect indication of the formation of the major metabolite AFBO and AFG_1_ was added into the reaction mixture as an internal standard. HPLC eluent was monitored by fluorescence (λ_excitation_ = 365 nm, λ_emission_ = 440 nm).

The recombinant pig CYP3A29 and its mutants were cloned and expressed as His_6_-tagged fusion protein in *Escherichia coli*. CYP3A29 WT with an apparent molecular mass of 60 kDa was obtained by affinity chromatography, and its purity exceeded 95% (Figure 1A, lane 4). The production of protein was estimated to be approximately 20.8 mg/L bacterial culture. Fe^2+^ ∙ CO vs. Fe^2+^ difference spectra of CYP3A29 and the mutants are shown in Figure 1B. The recombinant proteins had a strong absorbance around 450 nm and no absorbance peak at 420 nm, indicating that CYP3A29 WT and the mutants expressed in the *E. coli* membrane possessed the spectral characteristics of functional P450.

To assess whether the recombinant CYP3A29 had epoxidation activity towards AFB_1_, incubation of CYP3A29 with AFB_1_ was performed. Because of the strong activation towards AFB_1_, quail liver microsomes were used as a positive control to identify the AFB_1_ metabolites produced by CYP3A29 [37,38]. As shown in Figure 1C top, a major product peak appeared at *t*_R_ 12.2 min after AFB_1_ incubation with quail liver microsomes. It has been reported that almost all AFB_1_ are metabolized to AFBO by quail liver microsomes, which can be assessed by the formation of dihydrodiol-Tris [39]. Meanwhile, the product peak at *t*_R_ 12.2 min disappeared in the absence of Tris in a control experiment (data not shown). Using LC-MS/MS (Agilent 6410B Triple Quad system, Santa Clara, CA, USA, Figure S1), we further identified that the metabolite at *t*_R_ 12.2 min was a dihydrodiol–Tris conjugate (see the structure in Figure 1C). When CYP3A29 was incubated with AFB_1_, an identical peak was detected at *t*_R_ 12.2 min (Figure 1C, middle). Furthermore, in the presence of 5 μM ketoconazole, the product at *t*_R_ 12.2 min was lost (Figure 1C, bottom), indicating that the oxidizing activity of CYP3A29 to AFB_1_ was efficiently inhibited by the specific CYP3A inhibitor. These results indicated that recombinant pig CYP3A29 expressed in *E. coli* possessed substantial AFB_1_ oxidizing activity and was indeed capable of activating AFB_1_ in vitro.

### 2.2. The CD Spectra and Nifedipine-Oxidized Activities of CYP3A29 and Its Mutants

**Figure 2:**
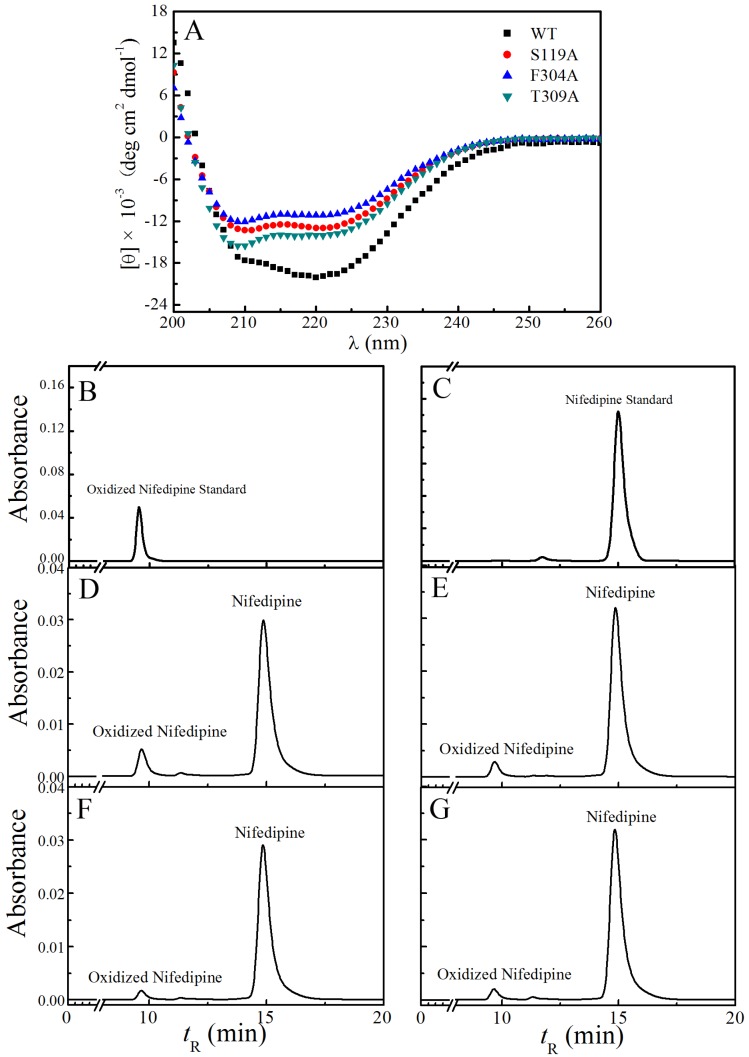
The circular dichroism spectra and CYP3A29 and the mutants’ nifedipine oxidation activities. (**A**) Far-UV CD spectra of CYP3A29 and its mutants. CD spectra were recorded at 20 °C and in 50 mM potassium phosphate buffer (200 mM KCl, 0.2 mM DTT, 1 mM EDTA, and 20% glycerol, pH 7.4). (**B**–**G**) The HPLC chromatogram of oxidized nifedipine standard; the HPLC chromatogram of nifedipine standard; HPLC chromatograms of nifedipine metabolized by WT, S119A, F304A, and T309A, respectively. HPLC eluent was monitored by absorbance at 254 nm.

To obtain information about the secondary structure of CYP3A29 and its mutants, we carried out circular dichroism experiments. The far-UV CD spectra of all the proteins displayed two negative peaks at 208 nm and 222 nm (Figure 2A), suggesting typical structures rich in α-helixes. The analysis by CONTINLL software (Colorado State University, Fort Collins, CO, USA, 2000) showed that the recombinant CYP3A29 contained 48.7% α-helix and 9% β-sheet (Table 1), which is similar to the contents of the secondary structure of human CYP3A4 (43% α-helix and 7% β-sheet, data from RCSB Protein Data Bank, 1W0E). The secondary structure contents (45.7% α-helix and 11% β-sheet, Table 1) of S119A were approximately the same as those of WT. Interestingly, compared with the other proteins, F304A contained the minimum α-helix (40.7%) and maximum β-sheet (13.9%), which was exactly the opposite to the case of T309A (52.8% α-helix, 7.9% β-sheet, Table 1).

Nifedipine was used as the specific substrate for CYP3A family to assess the activities of CYP3A29 and the mutants. The HPLC results displayed that nifedipine could be metabolized into oxidized nifedipine by all the enzymes after in vitro incubations, as confirmed by the product standard (Figure 2B). Native CYP3A29 exhibited the maximum oxidizing activity to nifedipine (Figure 2D), whereas S119A had only a half activity of the WT towards the model substrate (Figure 2E). F304A and T309A showed even weaker oxidation than S119A (Figure 2F,G). These results reflected the effect of specific residue on recognizing nifedipine.

### 2.3. Comparison of AFB_1_ Activation by CYP3A29 and Its Mutants

**Figure 3:**
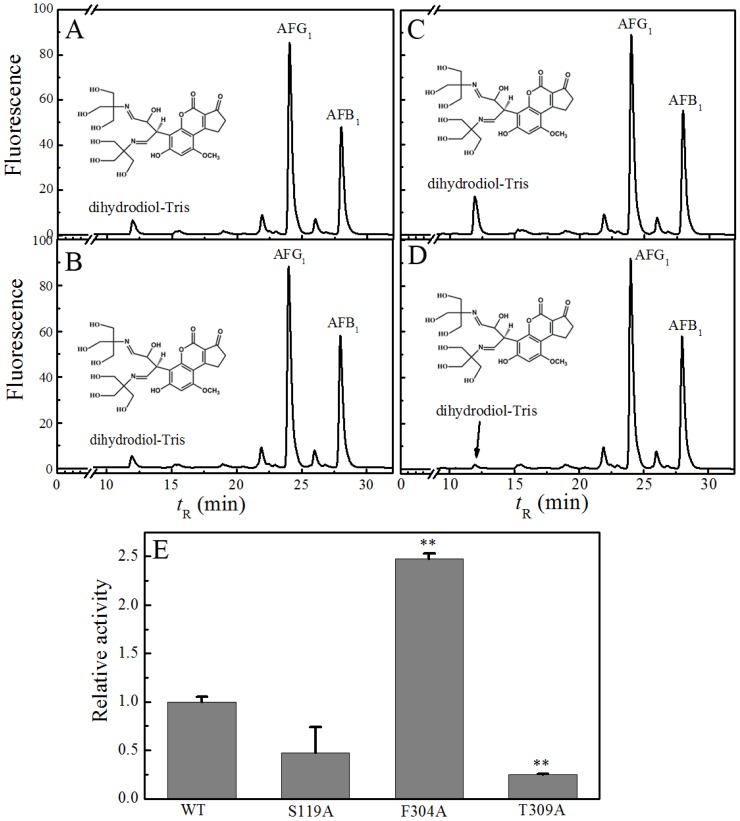
AFB_1_ oxidation by CYP3A29 and the mutants. (**A**–**D**) HPLC chromatograms of AFB_1_ metabolized by WT, S119A, F304A, and T309A, respectively; (**E**) histograms of relative activities of CYP3A29 and the mutants towards AFB_1_. The dihydrotriol–tris is an indirect indication of the formation of the major metabolite AFBO and AFG_1_ was added as an internal standard. HPLC eluent was monitored by fluorescence (λ_excitation_ = 365 nm, λ_emission_ = 440 nm). ** *p* < 0.01, Values are means ± SE (*n* = 3).

To explore the regioselectivity and specificity of pig CYP3A29 towards AFB_1_, we carried out the incubation of CYP3A29 and its mutants with AFB_1_. Oxidizing activities of all recombinant enzymes towards AFB_1_ can be relatively quantified and compared by analyzing the ratio of peak area of the main product to that of AFG_1_ (the original data was shown in Table S1). The relative activities of WT and its mutants can be seen in Figure 3E. Compared with WT (Figure 3A), the substitution of Ser119 with Ala resulted in the middle impairment (about 50%) of AFB_1_ exo-epoxidation (Figure 3B). Unexpectedly, replacement of Phe304 with Ala led to over 2-fold increase of AFB_1_ epoxidation activity (Figure 3C), which is totally opposite to the result observed in human CYP3A4 [36]. For Thr309, its substitution with Ala resulted in the significant impairment (about 80%) of AFB_1_ exo-epoxidation (Figure 3D), which implied that threonine at the position 309 may play an important role in the activation of AFB_1_.

### 2.4. Molecular Docking of AFB_1_ into CYP3A29 and the Mutants

CYP3A29 structure generated by SWISS-MODEL server was firstly evaluated by PROCHECK program, which gives a Ramachandran plot and a quantitative distribution of residues [41]. The percentage of residues in the most favored, allowed, and disallowed conformations were 78.1%, 21.4%, and 0.5%, respectively. The overall quality factor calculated by Errat was 74. And the averaged 3D-1D score passed the verification by Verify_3D.

**Figure 4:**
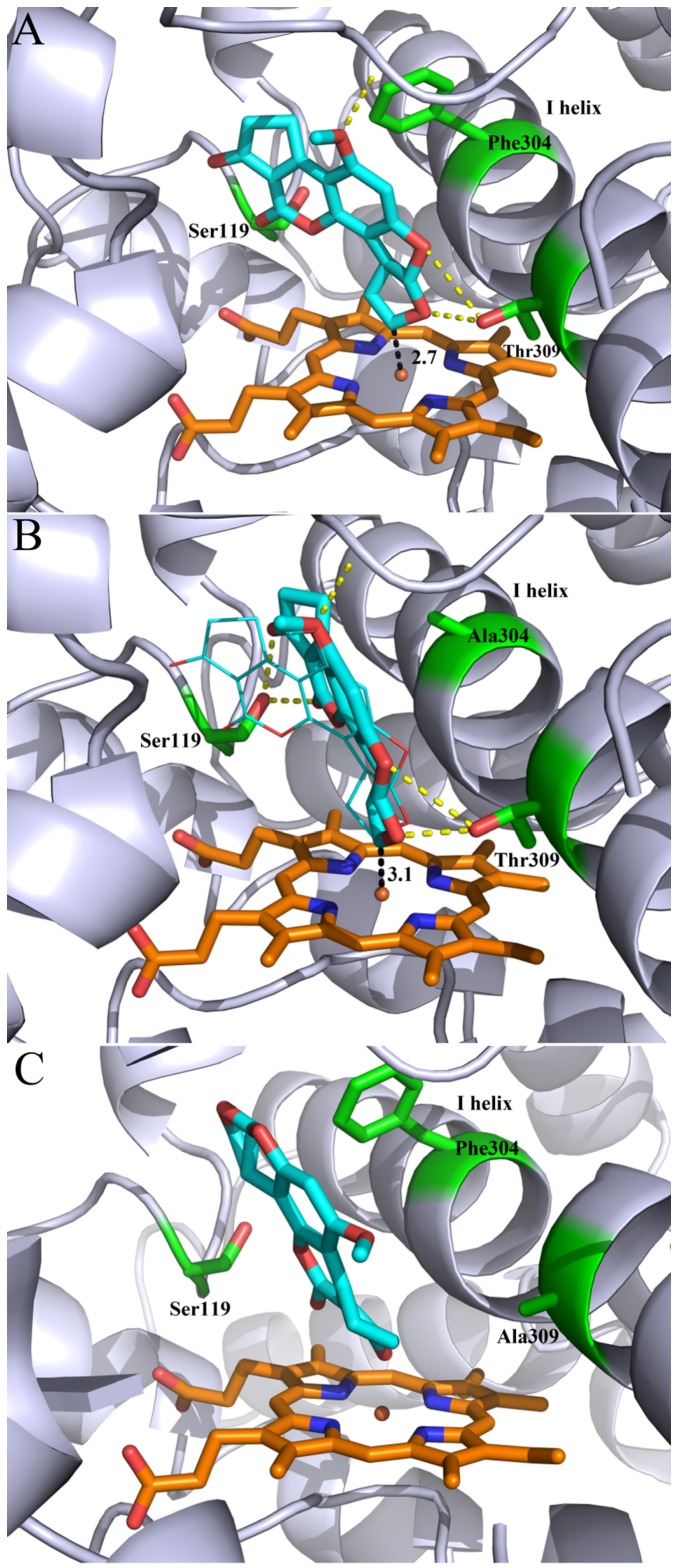
Molecular models of AFB_1_ docked into CYP3A29 and the mutants. (**A**) The docking conformation of AFB_1_ in the active site of CYP3A29; (**B**) the docking conformation of AFB_1_ in F304A. The best conformation of AFB_1_ was docked into F304A (shown as thick sticks), while that of AFB_1_ in WT is shown as thin sticks; (**C**) The docking conformation of AFB_1_ in T309A. CYP3A29 is shown in a ribbon format, and an iron ion is shown as a sphere. The examined amino acid residues are green, and the heme is orange. Color scheme: red for oxygen atoms and blue for nitrogen atoms. Hydrogen bonds are shown as yellow dashed lines, and distances between atoms are shown with black dashed lines and given in angstroms. Residues 368–371 and 476–484 are not shown, for clarity. The figures are rendered with PyMOL (Version 1.8, Schrodinger, LLC., New York, NY, USA, 2015) [42].

To establish a binding model and understand the details of interaction between ligand and receptor, we carried out the molecular docking study on AFB_1_ with CYP3A29 and its mutants. One hundred docking conformations generated by AutoDock 4.2 were clusters with an RMSD of 2.0 Å and ranked by the lowest docked energy. Two clusters were produced by the docking simulations when AFB_1_ was docked into receptor CYP3A29 WT, F304A, and S119A respectively, and only one for T309A. For WT, the first cluster contained 37% conformers and had −8.58 kcal/mol of the lowest binding energy (Table 2). It can be seen from the side view of the docking model that AFB_1_ was located to the upper right of the heme iron (Figure S2), in which orientation the epoxy bond could form only on the left of the terminal furan ring. Stereochemically, this is AFB_1_-exo-8,9-epoxide. Consistent with the previous reports [31,36], the docking only produced the orientation that was favorable for forming exo-8,9-epoxidation while no orientation for endo-8,9-epoxidation. In the first cluster, AFB_1_ almost stood vertically onto the heme, the redox center, with its C8 oriented towards the heme iron (Figure 4A). Furthermore, the measured interatomic distance between C8 and heme iron was 2.7 Å (Figure 4A), which allowed the contact of the ferric peroxy anion and led to the electrons’ transfer from the substrate and the subsequent formation of AFBO. Therefore, the conformations in this cluster predicted formation of active metabolite. For the other cluster, on the contrary, the docking result showed that the oxygen atom at C1 of AFB_1_ was oriented towards the heme iron, whereas C8, C9-double bond was far away from the oxidized center, in which case no AFBO could be formed, thus these conformations were thought inactive. It also can be seen from the putative binding model that AFB_1_ interacted with residues in the active site by forming two hydrogen bonds: one was between side chain hydroxyl group of Tyr309 and O6 or O7 of AFB_1_; the other was between the oxygen atom at C4 of AFB_1_ and the hydrogen in a peptide bond formed by Lys212 with Phe213 (Figure 4A). It is speculated that these two hydrogen bonds ought to play special roles in maintaining the stable binding of AFB_1_ in the active center.

When Phe304 was substituted with Ala, the orientational transformation of AFB_1_ molecule could be observed through the docking simulation. 87% conformers were organized into the first cluster, with −8.74 kcal/mol of the lowest binding energy (Table 2). Similar to the pose in WT, C8 of AFB_1_ was still directed towards the heme iron, with a interatomic distance of 3.1 Å (Figure 4B). However, AFB_1_ in F304A rotated clockwise for 42.1° compared with the pose in WT, turning into nearly parallel to I helix on the right side (Figure 4B). In this conformation, a new hydrogen bond was formed between the side chain hydroxyl group of Ser119 and carbonyl group oxygen at C1 or C11 of AFB_1_ (Figure 4B), which would further stabilize the binding of AFB_1_ in the active center.

For the replacement of Thr309 with Ala, interestingly, only one cluster was produced by the docking simulation. In all conformations within the cluster oxygen atom at AFB_1_ C1 was directed towards the heme iron (Figure 4C), suggesting no plausible oxidation product (Table 2). This result revealed that Thr309 may play a critical role in keeping the correct orientation of AFB_1_ because the loss of the hydrogen bond between aflatoxin and the amino acid resulted in the molecular inversion of AFB_1_ and formation of the inactive pose.

Considering that S119A mutation did not result in obvious alteration on both active conformation proportion (40%) and the lowest binding energy (−8.57 kcal/mol, Table 2) in comparison with the WT according to the docking results, it is unlikely that Ser119 is involved in direct interaction with AFB_1_.

## 3. Discussion

The key P450 oxidases responsible for the bioactivation of AFB_1_ in numbers of species or tissues have been well documented [22,23,24,43]. In pigs, four CYP3A isoforms, CYP3A22, CYP3A29, CYP3A39, and CYP3A46, have been identified so far, and characterized mainly in T-2 biotransformation [34,44,45,46]. The oxidation of testosterone and nifedipine by pig CYP3A29 has been well characterized [34]. However, there are no data on P450-mediated activation of AFB_1_ in pigs. In this study we focused on the cloning and characterization of CYP3A29, and investigation of the interaction of this enzyme with AFB_1_, demonstrating that CYP3A29 mediated conversion of aflatoxin to the toxic epoxide.

The recombinant CYP3A29 expressed in *E. coli* demonstrated a typical P450 spectral characteristic. The ordered secondary structure was confirmed by CD spectra. In the in vitro incubation experiment, to validate the activity of P450 oxidase, nifedipine, the CYP3A4 specific substrate was transformed into the oxidized form by purified CYP3A29, suggesting that the expressed recombinant enzyme has the capacity for biotransformation of compounds. In vitro incubation experiments further confirmed that CYP3A29 converts AFB_1_ to toxic epoxide AFBO. It should be noted that this activation was evidenced indirectly by the formation of a dihydrodiol–Tris conjugate in this study because of the instability and short half-life of the AFBO [47,48]. AFB_1_ epoxide easily undergoes hydrolysis at neutral solution and transforms into AFB_1_ 8,9-dihydrodiol [48], which reacts with Tris to form a highly fluorescent compound [49], thus greatly elevating the sensitivity and benefiting the detection. Therefore, the activation of AFB_1_ can be measured by the formation of a dihydrodiol–Tris adduct [50]. Quail liver microsomes were used as a positive control as they were reported to be highly active in AFB_1_ epoxidation [37,51,52,53]. The HPLC assays showed that the AFB_1_ metabolite produced by quail microsomes had the same retention time as that formed during CYP3A29 reaction with AFB_1_, suggesting that CYP3A29 was capable of oxidizing AFB_1_ into AFBO.

On the primary structure, CYP3A29 enzyme consists of 503 residues and is believed to be the homolog of human CYP3A4 because it shares 77% sequence identity with CYP3A4. Similar to human CYP3A4, CYP3A29 has a series of key amino acid residues that contribute to effector binding and cooperativity. For example, Leu-210 and Leu-211 are believed to be highly conserved in CYP3A family and contribute to effector binding [54], and Phe-304 is deemed to play dual roles in cooperativity, regioselectivity, and stereoselectivity [55,56,57]. To gain insight into the regioselectivity and substrate recognition of CYP3A29 towards AFB_1_, we mutated three residues located in substrate recognition sites, S119A, F304A, and T309A. Similar to the WT, the purified mutants possessed an ordered secondary structure that was rich in α-helix. Furthermore, in vitro reconstitution assays showed varied oxidizing activity to nifedipine in WT and mutants, suggesting that the mutants are functional in biotransformation of exogenous compounds, though these three mutations caused the deterioration of the oxidation of CYP3A29 to nifedipine to different extents. More importantly, they exhibited different metabolism to AFB_1_. Thr309 played an important role in orientation of AFB_1_, because removal of hydroxyl group of Thr309 severely impaired AFB_1_ epoxidation. The CYP3A29–AFB_1_ binding model deduced from molecular docking revealed that two hydrogen bonds would be formed between AFB_1_ and the surround residues in the active center, one of which was located between the hydroxyl group of Tyr309 and O6 or O7 of AFB_1_ (Figure 4A). This hydrogen bond seemed to be essential for the binding of AFB_1_ because any alternative active orientation could be obtained when AFB_1_ was docked into T309A (Figure 4C, Table 2). The data may explain the reason why the replacement of Thr309 with Ala resulted in a large loss of activity. It further supported the fact that Thr309 plays a significant role in binding and orientation of AFB_1_ by forming a hydrogen bond with it in the active pocket.

In contrast to the docking study for human CYP3A4, in which replacement of Phe304 with Ala caused a complete loss of AFB_1_ oxidation [36], the pig CYP3A29 F304A mutant exhibited enhanced activity to AFB_1_ epoxidation. The CYP3A4–AFB_1_ binding model deduced from molecular docking reveals that a strong π–π interaction between Phe304 phenyl group and the aromatic ring in AFB_1_ plays a key role in AFB_1_ binding [36]. However, we could not find the π–π interaction between Phe304 and AFB_1_ in the docking simulation of CYP3A29 (Figure 4A). Instead of the π–π interaction, our CYP3A29 docking model showed the side-chain phenyl group of Phe304 is a steric hindrance and, therefore, results in energetically unfavorable binding for AFB_1_ (binding energy, −8.55 kcal/mol, Table 2). We believe that in the mutant F304A, removal of the bulk side-chain phenyl group of Phe304 allows the ligand AFB_1_ molecule to be located closer to the I-helix and allows the formation of another hydrogen bond between the hydroxyl group of Ser119 and the oxygen atom at C1 or C11 in AFB_1_ which additionally stabilized AFB_1_. This is supported by the more proportional conformations (87%) and a lower binding energy (−8.74 kcal/mol, Table 2) obtained in the molecular docking, in comparison with those of WT. These results can explain why F304A exhibited enhanced activation towards AFB_1_ compared to WT.

The capacity of activating AFB_1_ was observed to be weakened but not significantly in the S119A mutant. However, the direct interaction between Ser119 and AFB_1_ could not be observed in the binding model deduced by molecular docking (Figure 4A). Meanwhile, AFB_1_ docking into S119A did not produce substantial differences compared to the WT, neither in the conformations nor the lowest energy (Table 2). It is reported that Ser119-mediated polar interactions play an important role in CYP3A4–ligand binding regardless of the hydrogen bond [58]. It is suggested that Ser119 might potentially contribute in mediating the AFB_1_ binding.

It should be pointed out that the interactions of these residues with AFB_1_ were deduced from molecular dockings because no CYP3A29 crystal structure was available. Allowing for the high sequence identity with human CYP3A4, it is supposed that the data would more likely be compatible with the authentic 3D model of CYP3A29, which calls for further validation in future study.

Based on previous studies and this research, it can be concluded that the regioselectivity of CYP3A29 towards different substrates is complicated and relatively difficult to predict. For instance, recent research suggests that Arg105, Arg106, Ser119, and Lys212 in CYP3A29 play important roles in the hydroxylation of the T-2 toxin [35]; many of them are not addressed in our study, with the exception of Ser119, which has only a slight and uncertain effect on AFB_1_ epoxidation. However, for the first time we have revealed that CYP3A29 is capable to oxidize AFB_1_ and it possibly has a different molecular mechanism to human CYP3A4. This is an example of the diversity of AFB_1_ recognition and probably other exogenous substrates for the evolutionarily conserved P450 isoforms.

## 4. Conclusions

In summary, this study clearly demonstrates that CYP3A29 is capable of activating AFB_1_ into AFBO. To our knowledge, this is the first report about pig CYP that involves AFB_1_ bioactivation. In addition, Thr309 in pig CYP3A29 plays a significant role in the stabilization of AFB_1_ binding in the active center through a hydrogen bond. Unlike human CYP3A4, removing the bulk side chain eliminates the steric hindrance of the phenyl group to the binding of AFB_1_, which enhances CYP3A29 epoxidation activity towards AFB_1_ in mutant F304A. Ser119 may mediate the binding of AFB_1_ by polar contact. Our study provides a reference for understanding the metabolic characteristics of AFB_1_ in pigs, which differs from well-studied CYP3A4 in human, and encourages further in vivo studies on the roles of CYP3A29.

## 5. Experimental Section

### 5.1. Ethics Statement

All the experiments involving the use of animals were conducted in accordance with the recommendations in the Regulations for the Administration of Affairs Concerning Experimental Animals of Guangdong province, China. This research was approved by Laboratory Animal Ethics Committee of South China Agricultural University, and the approval number was 2015-04. A Duroc × Yorkshire × Landrace crossbred pig (4–5 months old) and three quails were used from Animal Husbandry Institute, Guangdong Academy of Agricultural Sciences. The quail livers were isolated to make the microsomes. The animals were sacrificed by injection of excess sodium pentobarbital, and then the surgeries of liver separation were performed.

### 5.2. Materials

AFB_1_ was purchased from Fermentek (Jerusalem, Israel). E.Z.N.A^®^ Total RNA Kit was purchased from Omega Bio-Tek (Norcross, GA, USA). Prime Script RT reagent kit and restriction endonucleases were obtained from Takara BIO Inc. (Kyoto, Japan). High fidelity DNA polymerase KOD-Plus- and KOD FX were from Toyobo (Osaka, Japan). QuikChange site-directed mutagenesis kit was purchased from Stratagene (La Jolla, CA, USA). Isopropyl-β-d-thiogalactoside (IPTG) and NADPH were from Gen-View Scientific Inc. (Wellington, FL, USA). Nifedipine (>98% purity), oxidized nifedipine (~95% purity) standards, NADPH cytochrome P450 oxidoreductase (94% purity), and cytochrome b_5_ (≥90% purity) were purchased from Sigma-Aldrich Corporation (St. Louis, MO, USA). A 5-mL HisTrap HP column was purchased from GE Healthcare (Uppsala, Sweden). A ZORBAX SB-C_18_ column (4.6 × 250 mm, 5 μm) was purchased from Agilent Technologies (Palo Alto, CA, USA). Chromatographic grade acetonitrile, methanol, and formic acid were obtained from Merck (Darmstadt, Germany). All the primers were synthesized in Invitrogen Biotechnology (Guangzhou, China). All the other reagents and chemicals used were of analytical grade available.

### 5.3. Molecular Cloning of Pig CYP3A29

Total RNA was isolated from porcine liver using a Total RNA kit. The first-strand cDNA was synthesized from 2 μg of total RNA using a Prime Script RT reagent kit. According to the published mRNA sequence of pig *CYP3A29* (NCBI NM_214423), the forward primer 5′-GGAAAATCCGAGGAGAGAATCA-3′ and the reverse primer 5′-CATCAAAGCCCAAGTCCTTAGAG-3′ were designed to amplify the interest gene. PCR reaction was performed using a thermocycling program that ran for 2 min at 94 °C and then proceeded for 32 cycles at 98 °C for 10 s, 60 °C for 30 s, and 68 °C for 1.5 min. The PCR product was ligated into the pMD 19-T vector through TA cloning. The CYP3A29 gene sequence was verified by DNA sequencing. Subsequently, *CYP3A29* gene was subcloned into the pCW *Ori*^+^ expression vector. To allow functional expression in *E. coli*, the cDNA fragment encoding the bacterial *ompA* leader sequence were fused to *CYP3A29* by PCR in frame with the initiation codon [59]. The leader sequence was proteolytically removed during bacterial synthesis, thus releasing the native CYP3A29. To purify efficiently the recombinant protein, 6× His tag encoding sequence were appended to the 3′ terminus of *CYP3A29* gene. The resulting plasmid was confirmed by DNA sequencing and named pCW/3A29.

### 5.4. Site-Directed Mutagenesis

The pCW/3A29 expression vector was used as the plasmid DNA template for oligonucleotide-directed mutagenesis. According to our preliminary docking results and other related reports [36,55], the S119A, F304A, and T309A mutants were prepared using a QuikChange site-directed mutagenesis kit. The primers used for mutagenesis were 5′-CTATGAGAAACGCTCTCGCTCTGGCTGAGGATGA-3′ for S119A, 5′-CCAAGGTATTATTTTTATTGCTGCTGGCTATGAGAC-3′ for F304A, and 5′-TATTTTTGCTGGCTATGAGGCCACTAGCAGTGCTCT-3′ for T309A. All of the mutations were verified by DNA sequencing.

### 5.5. Protein Expression and Purification

Recombinant CYP3A29 and the mutants were expressed in *E. coli* DH5α cells. A single colony containing the recombinant plasmid was inoculated and cultured in 500 mL of TB broth containing 100 μg/mL ampicillin, 1 mM thiamine, and a mixture of trace elements. The cells were grown at 37 °C until the absorbance at 600 nm reached 0.8. Then, the expression was induced at 30 °C by the addition of 1 mM isopropyl β-d-thiogalactoside (IPTG). After 36 h, the cells were harvested and treated by 0.25 mg/mL of lysozyme. The techniques used for cell disruption, preparation of spheroplasts, and isolation of membranes were as described previously [60] with some modifications. The bacterial membrane fragments collected by ultracentrifugation were solubilized in ice-cold buffer A (100 mM potassium phosphate, 500 mM KCl, 10% glycerol, 0.2 mM dithiothreitol, 1 mM EDTA, 20 mM imidazole, pH 7.4) containing 0.5% CHAPS and 0.65% sodium cholate. Then the suspension was centrifuged with 20,000 rpm at 4 °C for 30 min, and the supernatant was filtered through 0.22 μm nylon membrane and loaded onto a 5 mL HisTrap HP column pre-equilibrated with buffer A. The recombinant protein was eluted with a linear gradient of 100 to 500 mM imidazole in buffer B (100 mM potassium phosphate, 500 mM KCl, 10% glycerol, 0.2 mM dithiothreitol, 1 mM EDTA, 500 mM imidazole, pH 7.4). The fractions were combined and dialyzed against the buffer (100 mM potassium phosphate, 500 mM KCl, 20% glycerol, 0.2 mM dithiothreitol, 1 mM EDTA, pH 7.4) at 4 °C for 24 h. Aliquots of the proteins were stored at −80 °C.

### 5.6. CO Difference Spectrum

Fe^2+^∙CO vs. Fe^2+^ difference spectra of the recombinant CYP3A29 and the mutants were collected as described previously [61] on a UV-2550 spectrophotometer (SHIMADZU, Kyoto, Japan). The concentrations of cytochromes P450 were calculated from the absorption change at 450 nm relative to that at 490 nm using the extinction coefficient ε_450–490_ = 91 mM^−1^∙cm^−1^ [61].

### 5.7. CD Spectroscopy

Far-UV CD spectra of the recombinant CYP3A29 and the mutants were collected on a Chirascan spectrometer (Applied Photophysics, Leatherhead, UK). The measurement parameters and methods employed were described as previously [62]. The contents of the secondary structure of proteins were estimated by CONTINLL algorithm [40], which gave the smallest RMSD.

### 5.8. Reconstitution of Nifedipine Oxidation by CYP3A29 and the Mutants

Reconstitution of a nifedipine-oxidized system followed the method of Shaw et al. [63] with some modifications. Briefly, the incubation mixture consisted of 0.05 μM recombinant CYP protein, 0.1 μM NADPH cytochrome P450 oxidoreductase, 0.1 μM cytochrome b_5_, 0.02 mg/mL liposomes (l-*α*-dilauroyl-*sn*-glycero-3-phosphocholine, l-*α*-diloleoyl-*sn*-glycero-3-phosphocholine, l-*α*-dilauroyl-*sn*-glycero-3-phosphoserine (1:1:1, *w*/*w*/*w* per milliliter), 0.2 mg/mL sodium cholate, 3 mM glutathione, 30 mM MgCl_2_, and 200 μM nifedipine in 100 mM potassium phosphate buffer (pH 7.4), making a final volume of 200 μL. The reaction was initiated by the addition of 1 mM NADPH (final concentration) and performed at 37 °C for 10 min, before being terminated by 2 mL of cold methylene chloride. After being added into 100 μL of 2 M sodium carbonate buffer (pH 10.5), the mixtures were centrifuged at 3000 *g* for 10 min. The combined organic layers were transferred and evaporated to dryness under a stream of high-pure nitrogen. The residue was redissolved in 150 μL of methanol and analyzed by HPLC. HPLC analyses were performed on a Waters Alliance system equipped with 2695 separation module. A symmetry reverse-phase C_18_ column was used to analyze the substrates nifedipine and their metabolites. The injection volume was 20 µL. An isocratic elution with 55% (*v*/*v*) methanol in water was applied to separate the substrates and the metabolites with a flow rate of 1 mL/min at 30 °C. Elution was monitored by the UV absorbance at 254 nm. The nifedipine and oxidized nifedipine standards were used as the controls to identify the substrate and the product.

### 5.9. Incubation of Recombinant CYP3A29 and the Mutants with AFB_1_

The recombinant CYP3A29 was incubated in the presence or absence of 5 μM ketoconazole with 0.1 μM NADPH cytochrome P450 oxidoreductase, 0.1 μM cytochrome b_5_, 0.02 mg/mL liposomes (l-*α*-dilauroyl-*sn*-glycero-3-phosphocholine, l-*α*-diloleoyl-*sn*-glycero-3-phosphocholine, l-*α*-dilauroyl-*sn*-glycero-3-phosphoserine (1:1:1, *w*/*w*/*w* per milliliter), 0.2 mg/mL sodium cholate, 3 mM glutathione, 30 mM MgCl_2_, 50 mM Tris, and 8 μM AFB_1_ in 100 mM potassium phosphate buffer (pH 7.4). Subsequently, the mutants S119A, F304A and T309A were incubated in the reconstitution system without ketoconazole. The reaction was initiated by the addition of 1 mM NADPH (final concentration) and performed at 37 °C for 3 h prior to termination by addition of 200 μL ice-cold methanol spiked with 0.76 μM aflatoxin G_1_ as an internal standard. The samples were kept at −20 °C overnight to facilitate protein precipitation and then centrifuged at 10,000 *g* for 15 min prior to analysis [22,64]. The supernatants were filtered through a 0.2 μm nylon membrane before being injected into the HPLC. AFB_1_-8,9-dihydrodiol generated in this incubation system reacted with Tris to form a highly fluorescent compound [49]. Quail liver microsomes were made and used as a positive control to determine the peak retention time of the dihydrodiol–Tris conjugate [37,38]. The major metabolite was identified by LC/MS.

### 5.10. Detection of AFB_1_ Metabolites by HPLC

The AFB_1_ metabolites produced by CYP3A29 and the mutants were detected on a Waters Alliance e2695 liquid chromatography system (Waters Corporation, Milford, MA, USA) equipped with a 2475 fluorescence detector. Samples were separated at 25 °C on a ZORBAX SB-C_18_ column. The mobile phase, the gradient schedule, and detection condition were described as previously [62]. The flow rate was set to 1 mL/min, and the injection volume was 40 μL.

### 5.11. Homology Modeling and Molecular Docking

Because of the high sequence identity of CYP3A29 with human CYP3A4 (77%), the three-dimensional structure model of CYP3A29 was built using SWISS-MODEL server as a homology modeling tool [65,66,67], based on the X-ray crustal structure of human CYP3A4 [68]. To retain the reliability of the model, the hydrophobic NH_2_-terminal region of CYP3A29 (residues 3 to 24) was deleted. The quality of the resultant model structure was analyzed and evaluated by PROCHECK, What_check, Verify_3D, Errat, and Prove software (The Structure Analysis and Verification Server, Version 4, University of California, Los Angeles, LA, 2012).

A molecular docking study was conducted to construct a binding model of AFB_1_ to CYP3A29. The AutoDock 4.2 docking program was used to find the best configurations for receptor and ligand complex [69]. For each simulation, CYP3A29 receptor was kept rigid, while AFB_1_ ligand was flexible, in which only one bond was rotatable. To cover the whole active site of CYP3A29, the grid box was set to 60 × 60 × 60 points with spacing of 0.375 Å. The box center was set at 60.055, 76.691, and 9.028. AutoDockTools 1.5.6 (The Scripps Research Institute, La Jolla, CA, USA) was used to add Gasteiger charges to the CYP3A29 coordinates and AFB_1_. The Lamarckian genetic algorithm was used to determine the globally optimized conformations and orientations of the ligand with minimized free energy. All the docking simulations were performed with 100 cycles of running using an initial population size of 150 and a maximum of 2.5 million energy evaluations. The other parameters were set at their default values. Docking results were clustered using a root mean square deviation (RMSD) of 2.0 Å and were ranked according to the change in free energy (ΔG) estimated by AutoDock. Conformations with the lowest free energies of binding were selected as the best binding modes. Molecular graphics were generated by PyMOL [42].

### 5.12. Statistical Analysis

Resulting data are reported as the mean and standard error of means (Mean ± SE) based on at least three independent experiments. Statistical significance of differences between means was assessed by two-tailed *t* test and *p* < 0.05 was considered significant.

## Figures and Tables

**Table 1:** Contents of secondary structure elements of CYP3A29 and its mutants. CD spectra were analyzed by CONTINLL with a 56-protein Reference [40].

Samples	α-Helix	β-Sheet	Turns	Unordered
-	-	%		-
WT	48.7	9	16.2	26.1
S119A	45.7	11	17.7	25.5
F304A	40.7	13.9	18.4	27
T309A	52.8	7.9	14.9	24.3

**Table 2:** Conformation distributions and the lowest binding energy of AFB_1_ docked into CYP3A29 and its mutants.

Protein	Active *	Inactive	Lowest Binding Energy ** (kcal/mol)
WT	37%	63%	−8.55
S119A	40%	60%	−8.57
F304A	87%	13%	−8.74
T309A	0	100%	-

* Percentages were obtained by calculating the ratio of active or inactive conformation quantity to 100 conformations; ** Only point to the energy in active conformation.

## Supplementary Materials

The following are available online at www.mdpi.com/2072-6651/8/9/267/s1, Figure S1: The identification of metabolite by LC-MS/MS; Figure S2: The side view of docking model; Table S1: The relative quantification of metabolic activities of CYP3A29 and its mutants to AFB_1_.

## Abbreviations

The following abbreviations are used in this manuscript:

AFB_1_	aflatoxin B_1_
CYP	Cytochrome P450
AFG_1_	aflatoxin G_1_
AFBO	AFB_1_ exo-8,9-epoxide
WT	wide-type
IPTG	isopropyl-β-d-thiogalactoside
CHAPS	3-[(3-cholamidopropyl)dimethylammonio]-1-propanesulfonate
EDTA	ethylenediaminetetraacetic acid
NADPH	nicotinamide adenine dinucleotide phosphate
NCBI	National Center for Biotechnology Information
HPLC	High-performance liquid chromatography
CD	circular dichroism
RMSD	root mean square deviation
BHA	butylated hydroxyanisole
