# Supplementary material for: Bioactivation and Regioselectivity of Pig Cytochrome P450 3A29 towards Aflatoxin B1
Source: Toxins (Basel). 2016 Sep 12;8(9):267. doi: 10.3390/toxins8090267 (PMC5037493; doi:10.3390/toxins8090267)
Supplement: Supplementary file 1 [file toxins-08-00267-s001.pdf]

Supplementary Materials: Bioactivation and Regioselectivity of Pig Cytochrome P450 3A29 towards Aflatoxin B_1

Jun Wu, Ruohong Chen, Caihui Zhang, Kangbai Li, Weiying Xu, Lijuan Wang, Qingmei Chen, Peiqiang Mu, Jun Jiang, Jikai Wen and Yiqun Deng

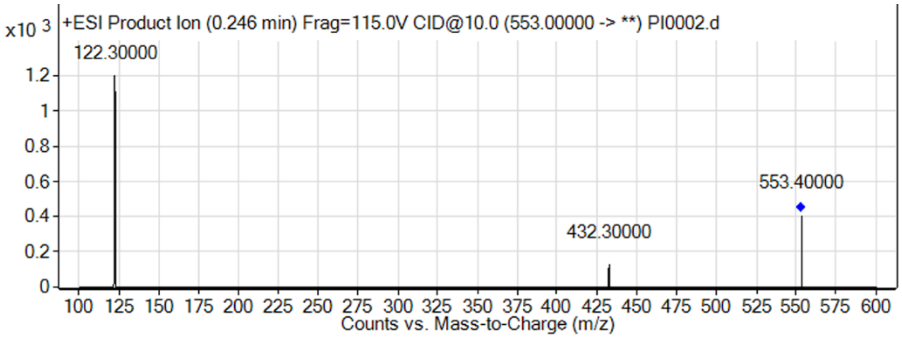

Figure S1. The identification of metabolite by LC-MS/MS.

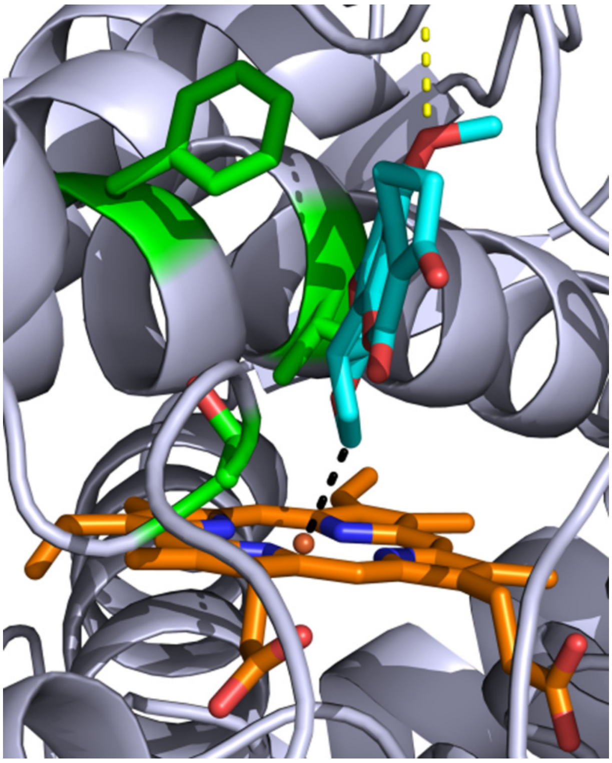

Figure S2. The side view of docking model.

Table S1. The relative quantification of metabolic activities of CYP3A29 and its mutants to AFB_1.

Enzymes	Dihydrodiol-Tris	AFG_1	Dihydrodiol-Tris/AFG_1	Normalized Values *
WT1	1,775,720	19,910,728	0.089	-
WT2	1,970,378	20,402,016	0.097	-
WT3	1,600,007	18,956,344	0.084	1.00 (0.090)
S119A1	403,518	21,918,143	0.018	-
S119A2	1,319,961	19,772,791	0.067	-
S119A3	504,333	12,758,786	0.040	0.46 (0.042)
F304A1	3,506,055	15,406,563	0.23	-
F304A2	4,335,512	19,841,307	0.22	-
F304A3	3,913,793	18,719,767	0.21	2.42 (0.22)
T309A1	120,478	5,361,935	0.022	-
T309A2	467,321	20,436,934	0.023	-
T309A3	456,039	20,808,153	0.022	0.25 (0.0224)

* The values in brackets represent the average of three repeats.
